# Changes in the transcriptome of the malaria parasite *Plasmodium falciparum* during the initial phase of transmission from the human to the mosquito

**DOI:** 10.1186/1471-2164-14-256

**Published:** 2013-04-15

**Authors:** Che Julius Ngwa, Matthias Scheuermayer, Gunnar Rudolf Mair, Selina Kern, Thomas Brügl, Christine Clara Wirth, Makoah Nigel Aminake, Jochen Wiesner, Rainer Fischer, Andreas Vilcinskas, Gabriele Pradel

**Affiliations:** 1 Research Center for Infectious Diseases, University of Würzburg, Josef-Schneider-Straße 2/D15, 97080 Würzburg, Germany; 2 Instituto de Medicina Molecular, Faculdade de Medicina da Universidade de Lisboa, Av. Professor Egas Moniz, Lisboa 1649-028, Portugal; 3 Institute of Molecular Biotechnology, RWTH Aachen University, Worringerweg 1, 52074 Aachen, Germany; 4 Fraunhofer Institute for Molecular Biology and Applied Ecology IME, Bioresources Project Group, Winchesterstraße 2, 35394 Gießen, Germany; 5 Institute of Phytopathology and Applied Zoology, University of Gießen, Heinrich-Buff-Ring 26-32, 35392 Gießen, Germany

**Keywords:** Malaria, *Plasmodium falciparum*, Gametocyte, Gametogenesis, Transmission, Mosquito, Transcriptome

## Abstract

**Background:**

The transmission of the malaria parasite *Plasmodium falciparum* from the human to the mosquito is mediated by dormant sexual precursor cells, the gametocytes, which become activated in the mosquito midgut. Because gametocytes are the only parasite stages able to establish an infection in the mosquito, they play a crucial role in spreading the tropical disease. The human-to-mosquito transmission triggers important molecular changes in the gametocytes, which initiate gametogenesis and prepare the parasite for life-cycle progression in the insect vector.

**Results:**

To better understand gene regulations during the initial phase of malaria parasite transmission, we focused on the transcriptome changes that occur within the first half hour of parasite development in the mosquito. Comparison of mRNA levels of *P. falciparum* gametocytes before and 30 min following activation using suppression subtractive hybridization (SSH) identified 126 genes, which changed in expression during gametogenesis. Among these, 17.5% had putative functions in signaling, 14.3% were assigned to cell cycle and gene expression, 8.7% were linked to the cytoskeleton or inner membrane complex, 7.9% were involved in proteostasis and 6.4% in metabolism, 12.7% were cell surface-associated proteins, 11.9% were assigned to other functions, and 20.6% represented genes of unknown function. For 40% of the identified genes there has as yet not been any protein evidence.

For a subset of 27 genes, transcript changes during gametogenesis were studied in detail by real-time RT-PCR. Of these, 22 genes were expressed in gametocytes, and for 15 genes transcript expression in gametocytes was increased compared to asexual blood stage parasites. Transcript levels of seven genes were particularly high in activated gametocytes, pointing at functions downstream of gametocyte transmission to the mosquito. For selected genes, a regulated expression during gametogenesis was confirmed on the protein level, using quantitative confocal microscopy.

**Conclusions:**

The obtained transcriptome data demonstrate the regulations of gene expression immediately following malaria parasite transmission to the mosquito. Our findings support the identification of proteins important for sexual reproduction and further development of the mosquito midgut stages and provide insights into the genetic basis of the rapid adaption of *Plasmodium* to the insect vector.

## Background

Up to date, the tropical disease malaria is one of the most devastating infectious diseases in the world, causing 216 million new cases and approximately 655.000 casualties each year [1]. Disease treatment and control measures are undermined by the spread of drug resistance in malaria parasites, particularly in populations of *Plasmodium falciparum*, the agent responsible for malaria tropica (reviewed in [2]).

The transmission of malaria parasites from the human to the mosquito is mediated by specialized sexual precursor cells, the intraerythrocytic gametocytes. Maturation of *P. falciparum* gametocytes from stage I to stage V takes approximately 10 days, and during this period the gametocytes maintain a stable cell cycle arrest (reviewed in [3,4]). The mature gametocytes circulate in the human’s blood stream, but remain dormant until they are taken up by a blood-feeding mosquito.

When entering the mosquito midgut together with the blood meal, the gametocytes become activated from the dormant stage by external stimuli, i.e. a drop in temperature and the contact with the mosquito-derived molecule xanthurenic acid (XA) (reviewed in [5,6]). Gametocyte activation leads to rounding up of the cell, followed by parasite egress from the enveloping erythrocyte, which involves the rupture of two membranes, the parasitophorous vacuole membrane (PVM) and the erythrocyte membrane [7] (reviewed [8]). During gametogenesis the microgametocyte replicates its genome three times in order to produce eight motile microgametes. Following the fusion of micro- and macrogametes a zygote forms and develops into an infective ookinete within the following 24 hours. The motile ookinete possesses an apical complex which enables it to traverse the midgut epithelium before settling down and forming an oocyst between epithelium and basal lamina (reviewed in [5]).

During gametocytogenesis, *P. falciparum* expresses a new set of genes important for sexual development [9-14]. Ingestion by the blood-feeding mosquito again triggers molecular changes in the sexual stage parasites, which help adjusting the gametocytes to the insect and which on the one hand initiate sexual reproduction and further development of the parasite in the vector, on the other hand prepare the emerging gametes for the hostile environment of the mosquito midgut. Noteworthy, the midgut stages have to persevere outside a host cell for more than one day. During this time period, the cells are highly vulnerable to the aggressive factors of the gut, which among others include bacteria as well as human immune cells, antibodies and complement proteins present in the blood meal, and this exposure results in an approximate 1000-fold loss of parasite abundance [15] (reviewed in [5,6]).

Gametocyte maturation and gametogenesis are particularly accompanied by the coordinated expression of numerous adhesive surface proteins, including the EGF domain-containing proteins Pfs25 and Pfs28, the 6-cys proteins Pfs230 and Pfs48/45, and the LCCL domain-containing PfCCp proteins. It is noteworthy that the majority of these proteins can be divided into two classes: One class of the adhesion proteins, including Pfs230, Pfs48/45 and the six PfCCp proteins, is expressed within the parasitophorous vacuole (PV) of the developing gametocyte, and the majority of these proteins assemble to multimeric protein complexes [16,17] (reviewed in [18]). The adhesion proteins are subsequently present on the gamete surface, but expression of these proteins usually ceases during fertilization. The expression of the second class of surface proteins starts following parasite transmission to the mosquito, as was shown for Pfs25 and Pfs28, and expression often persists until the ookinete has formed (reviewed in [5]). One reason for this sudden onset of protein expression following gametocyte activation in the mosquito midgut is the translational repression of messenger RNA (mRNA) encoding for some of these proteins. This was inter alia shown in the rodent malaria parasite *P. berghei* for the repression of Pbs25 and Pbs28 by the RNA helicase DOZI (development of zygote inhibited) as part of a ribonucleoprotein complex [19]. DOZI and its subsequently identified interaction partner CITH (CAR-I and fly trailer hitch) are considered repressors of maternally supplied mRNA important for ookinete development [20].

While astonishing advances have been made in previous years in unveiling the molecular basis of parasite transmission from the mammalian host to the mosquito, the currently available data are fragmented and major key players for gametogenesis and adaptation have not yet been identified. We thus aimed at investigating the changes in transcript levels during gametocyte activation in order to gain in-depth knowledge on the molecular switch-over that takes place in the parasite during transmission. We selected the suppression subtractive hybridization (SSH) technique to experimentally screen for genes differentially expressed during gametocyte activation, because this PCR-based method amplifies induced genes while simultaneously suppressing house-keeping genes, and is thus particularly suited to discover new or unexpected genes [21]. Our data provide insights into the regulated gene expression of malaria parasites during transmission to the mosquito and enable the identification of novel proteins with essential functions for the mosquito midgut stages, which might be suitable targets for transmission blocking interventions.

## Results and discussion

To determine changes in the transcriptome of malaria parasites during the initial phase of transmission to the mosquito, we conducted a SSH on mRNA isolated from mature non-activated gametocytes and from gametocytes at 30 min after in vitro activation, which have completed gametogenesis [7]. A subtracted cDNA library was constructed and the resulting cDNA was assigned to the respective plasmodial genes.

**Figure 1:**
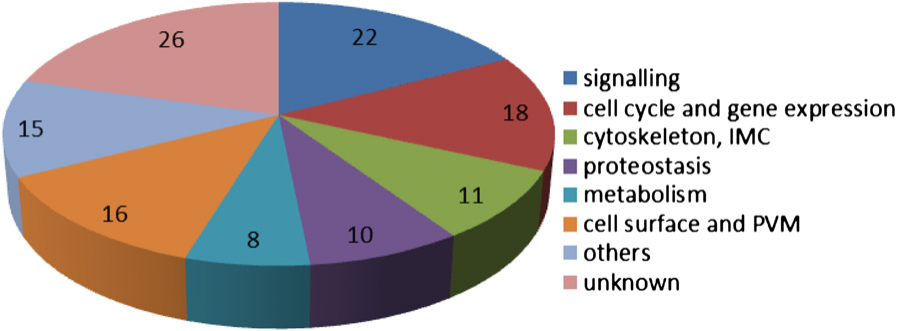
**Ontology groups of *P. falciparum *genes with changes in transcript expression during gametocyte activation.** The changes in transcript abundance were determined by SSH between the mRNA of mature non-activated gametocytes and gametocytes at 30 min p.a. in vitro. A total number of 126 genes were identified (the numbers of genes per group are indicated).

We identified a total number of 126 genes, for which expression levels changed in the gametocytes during activation. The majority of genes can be assigned to six major ontology groups (Figure 1, Table 1): 22 genes (17.5%) have putative functions in signaling, 18 genes (14.3%) are assigned to processes linked to cell cycle and gene expression, 11 genes (8.7%) can be linked to the cytoskeleton or the inner membrane complex (IMC), 10 genes (7.9%) have putative functions in protein trafficking, stabilization and degradation (proteostasis), eight genes (6.4%) are linked to general metabolic functions, and 16 genes (12.7%) are proteins of the cell surface or PVM. Furthermore, 15 genes (11.9%) can be assigned to other functions, including protein synthesis and processing. A total of 26 genes (20.6%) encode for proteins with unknown function, eight of which have sequences for transmembrane domains and five of which encode for a signal peptide.

**Table 1:** **Genes of the *Plasmodium falciparum *gametocyte transcriptome as identified by SSH (comparing mature non-activated gametocytes and gametocytes at 30 min p.a.)**

**Plasmodb ID**	**Protein**	**Predicted features**	**pred. MW**
**Signaling**			
PF3D7_0214600	serine/threonine protein kinase		204.3
PF3D7_1356800	serine/threonine protein kinase ARK3		475.7
PF3D7_0302100	serine/threonine protein kinase PfCLK-4/SRPK1	Phosphorylation of SR splicing factors	157.4
PF3D7_1230900	atypical protein kinase, RIO family		111.5
PF3D7_1238900	calcium/calmodulin-dependent protein kinase, PK2		58.9
PF3D7_0624000	hexokinase, HK		55.3
PF3D7_0515300	phosphatidylinositol 3-kinase, PI3K		255.9
PF3D7_1113100	protein tyrosine phosphatase, PRL		25.1
PF3D7_1403900	phosphatase		35.2
PF3D7_1464600	phosphatase	SP	170.5
PF3D7_0210600	conserved Plasmodium protein	SP, calcium-lipid-binding domain CaLB	53.7
PF3D7_1238100	calcyclin binding protein	Calcyclin-binding domain	266.4
PF3D7_1442200	GTP binding protein		118.7
PF3D7_1358600	zinc finger protein	Zinc-Finger domain	118.3
PF3D7_0927200	zinc finger protein	Zinc-Finger domain	189.7
PF3D7_1004300	zinc finger, C3HC4 type		131.7
PF3D7_1433400	conserved Plasmodium membrane protein	7 TMs, PHD C4HC3 zinc-finger-like motifs	682.5
PF3D7_1463900	conserved Plasmodium membrane protein	SP, 7 TMs, EF hand calcium-binding domain	127.1
PF3D7_1233600	asparagine and aspartate rich protein 1, AARP1	Zinc-finger domain, Ring/U-Box	146.3
PF3D7_0530200	triose phosphate transporter iTPT	SP, 7 TMs	59.8
PF3D7_0303900	conserved Plasmodium protein	SP, phosphatidyl-ethanolamine-binding protein PEBP domain	23.2
PF3D7_0818200	14-3-3 protein	Regulative protein, binds signaling enzymes like kinases or TM receptors	30.2
**Cell cycle and gene expression**			
PF3D7_1445900	DEAD/DEAH box ATP-dependent RNA helicase		60.0
PF3D7_1019000	eukaryotic translation initiation factor subunit eIF2A		280.8
PF3D7_1417200	NOT family protein	Regulation of transcript	519.8
PF3D7_1103800	CCR4-NOT transcription complex subunit 1, NOT1	4 TMs	392.0
PF3D7_0622900	ApiAP2 protein	Transcription factor	237.4
PF3D7_0420300	ApiAP2 protein	Transcription factor	400.2
PF3D7_0615400	ribonuclease		323.2
PF3D7_0811300	CCR4-associated factor 1, CAF1		200.2
PF3D7_1034900	methionine-tRNA ligase		101.2
PF3D7_1469700	conserved Plasmodium protein	Homology to the transcriptional mediator subunit Med6	24.6
PF3D7_0513600	deoxyribodipyrimidine photolyase		129.2
PF3D7_1105500	centrin-4 (CEN4)		20.1
PF3D7_1018500	PHF5-like protein	mRNA splicing factor RDS3 domain	12.5
PF3D7_0827800	SET domain protein SET3	Chromatin regulator, nuclear protein, in male gametocytes	283.6
PF3D7_1115200	SET domain protein, SET7	Chromatin regulator	94.3
PF3D7_1360900	polyadenylate-binding protein	RNA-binding domain	44.8
PF3D7_1011800	QF122 antigen	KH domain type 1, RNA binding	131.6
PF3D7_1347500	DNA/RNA-binding protein Alba 4, ALBA4		42.2
**Cytoskeleton, IMC, Motor complex**			
PF3D7_0422300	alpha-tubulin II	Cytoskeletal element	49.7
PF3D7_1246200	actin I	IMC component	41.2
PF3D7_0503400	actin-depolymerizing factor 1, ADF1		13.7
PF3D7_1020200	kinesin		99.1
PF3D7_0724900	kinesin-like protein		222.6
PF3D7_1246400	myosin A tail domain interacting protein, MTIP	IMC component	23.5
PF3D7_1251200	coronin	Actin-binding protein	69.0
PF3D7_0708000	cytoskeleton-associated protein	Cap-Gly domain	103.6
PF3D7_0515700	glideosome-associated protein 40, GAP40	10 TMs, IMC component	51.8
PF3D7_0918000	glideosome-associated protein 50, GAP50	SP, 2 TMs, IMC component	44.6
PF3D7_1351700	Alveolin, ALV6	IMC component	151.2
**Proteostasis (Protein trafficking, stabilization and degradation)**			
PF3D7_0807500	proteasome subunit alpha type 6	Component oft he proteasome core particle	29.5
PF3D7_1466300	proteasome subunit RPN2	Proteasome non-ATPase regulatory subunit 1	132.8
PF3D7_0815800	vacuolar sorting protein, VPS9	Ortholog of *Saccharomyces cerevisae* vacuolar sorting protein 9	214.7
PF3D7_1136400	conserved *Plasmodium* protein	TPR domain, mediates protein interactions and multi-protein complexes	107.1
PF3D7_0215400	conserved Plasmodium protein	WD40-domain protein, assembly and stabilization of MPCs	110.8
PF3D7_0818900	heat shock protein 70, HSP70-1		73.9
PF3D7_0917900	heat shock protein 70, HSP70-2		72.4
PF3D7_0306200	activator of Hsp90 ATPase, AHA1		41.6
PF3D7_0708400	heat shock protein 90, HSP90		86.2
PF3D7_0102200	ring-infected erythrocyte surface antigen, RESA	PHIST and DnaJ domains	126.5
**Metabolism, general**			
mal_mito_2	cytochrome c oxidase subunit I, cox1	SP, 12 TMs, component of the respiratory chain in mitochondria	53.0
PF3D7_1444800	fructose 1,6-biphosphate aldolase	Glycolysis pathway	40.1
PF3D7_0806800	vacuolar proton translocating ATPase subunit A	6 TMs	123.0
PF3D7_0922200	S-adenosylmethionine synthetase, SAMS		44.8
PF3D7_0801900	conserved Plasmodium protein	FAD/NADP-binding domain	552.0
PF3D7_0403200	conserved Plasmodium protein	Possibly acetyl-CoA-synthetase	69.4
PF3D7_0511800	myo-inositol 1-phosphate synthase		69.1
PF3D7_0104400	4-hydroxy-3-methylbut-2-enyl diphosphate reductase, LytB	1 TM, protein export domain, non-mevalonate pathway	62.5
**cell surface and PVM**			
PF3D7_1035300	glutamate-rich protein; GLURP	SP, on parasite surface	141.1
PF3D7_1335300	reticulocyte binding protein 2, RH2b	1 TM, on parasite surface, involved in erythrocyte binding	382.9
PF3D7_1228600	merozoite Surface Protein 9, MSP-9	SP, on merozoite surface, involved in erythrocyte binding	86.6
PF3D7_1028700	merozoite TRAP-like protein, MTRAP	SP, 1 TM, belongs to TRAP family, in micronemes and on merozoite surface	58.1
PF3D7_0731500	erythrocyte binding antigen 175, EBA-175	SP, 1 TM, Duffy binding domain, on merozoite surface	174.6
PF3D7_0102500	erythrocyte binding antigen-181, EBA-181	SP, 2 TMs, Duffy binding domain, on merozoite surface	181.2
PF3D7_1035700	duffy binding-like merozoite surface protein, DBLMSP	SP, Duffy binding domain, SPAM	80.3
PF3D7_0202500	early transcribed membrane protein 2, ETRAMP2	SP, 1 TM, integral PVM protein	11.5
PF3D7_0423700	early transcribed membrane protein 4, ETRAMP4	SP, 2TMs, integral PVM protein	10.2
PF3D7_1033200	early transcribed membrane protein 10.2, ETRAMP 10.2	SP, 2TMs, integral PVM protein	38.9
PF3D7_1103500	CPW-WPC family protein	SP, adhesin, cysteine-rich CPW-WPC domain	67.3
PF3D7_0406200	Pfs16	SP, 2 TMs, integral PVM protein of gametocytes	16.7
PF3D7_0930300	merozoite surface protein 1, MSP1	SP, GPI anchor, EGF domain, on merozoite surface, part of MSP complex	195.7
PF3D7_1218800	conserved Plasmodium protein, PSOP17	SP, lectin domain, laminin G2 domain	39.6
PF3D7_0707300	rhoptry-associated membrane antigen, RAMA	GPI anchor, involved in erythrocyte binding	103.6
PF3D7_1021900	10b antigen	Ankyrin repeat region, PHAX RNA-binding domain, interacts with surface proteins like PfEMP1 (Y2H)	267.4
**Others**			
PF3D7_1103100	60S ribosomal protein P1		13.0
PF3D7_1027800	60S ribosomal protein L3		44.2
PF3D7_1408600	40S ribosomal protein S8e		25.1
PF3D7_1358800	40S ribosomal protein S13		17.3
PF3D7_0206200	metabolite/drug transporter	11 TMs, Major facilitator superfamily	62.7
PF3D7_1207700	blood stage antigen 41–3 precursor	SP	43.4
PF3D7_1457000	signal peptide peptidase, SPP	8 TMs	47.6
PF3D7_0320700	signal peptidase complex subunit 2, SPC2	2 TMs, SPC25 domain	21.0
PF3D7_0207700	serine repeat antigen 4, SERA-4	SP, papain family cysteine proteinase	108.7
PF3D7_1033800	plasmepsin VII	SP, 1 TM, aspartyl protease	52.3
PF3D7_0930000	procollagen lysine 5-dioxygenase	2 TMs	66.8
PF3D7_0817600	conserved Plasmodium protein	MAC/PF domain	112.2
PF3D7_0403800	conserved Plasmodium protein	Alpha/beta-hydrolase	83.4
PF3D7_0709900	conserved Plasmodium membrane protein	4 TMs, alpha/beta-hydrolase	274.0
PF3D7_0311600	conserved Plasmodium protein, unknown function	SP, 1 TM, ribophorin-I domain,putative dolichyl-diphospho-oligosaccharide-protein	84.0
**Unknown**			
PF3D7_0904200	conserved Plasmodium protein	SP, 1 TM	34.0
PF3D7_1024800	conserved Plasmodium protein	SP, 2 TMs	171.1
PF3D7_1239400	conserved Plasmodium protein	SP	22.0
PF3D7_0730800.1	Plasmodium exported protein	SP	32.1
PF3D7_1316700	conserved Plasmodium protein	SP	73.4
PF3D7_0601900	conserved Plasmodium protein	1 TM	14.7
PF3D7_0704100	conserved Plasmodium membrane protein	6 TMs	425.3
PF3D7_1023000	conserved Plasmodium membrane protein	5 TMs	48.9
PF3D7_1439600	conserved Plasmodium protein	1 TM	22.6
PF3D7_1021700	conserved Plasmodium membrane protein	4 TMs	830.2
PF3D7_0417400	conserved Plasmodium protein	2 TMs, interacts with PF3D7_1021700 (Y2H)	822.4
PF3D7_1026600	conserved Plasmodium protein	Interacts with RNA-associated proteins (Y2H)	196.5
PF3D7_1362600	conserved Plasmodium protein	Interacts with splicing factor 3B subunit 2 (Y2H)	32.1
PF3D7_0202400	conserved Plasmodium protein	Pfg27 domain	141.9
PF3D7_1124200	conserved Plasmodium protein	UAS domain	49.3
PF3D7_1452400	conserved Plasmodium protein		107.5
PF3D7_1225600	conserved Plasmodium protein		93.1
PF3D7_1451200	conserved Plasmodium protein		176.2
PF3D7_1438800	conserved Plasmodium protein		84.0
PF3D7_1114600	conserved Plasmodium protein		39.6
PF3D7_1026100	conserved Plasmodium protein		23.9
PF3D7_1230100	conserved Plasmodium protein		61.5
PF3D7_1321000	conserved Plasmodium protein		80.6
PF3D7_0411000	conserved Plasmodium protein		179.7
PF3D7_1126700	conserved Plasmodium protein		110.1
PF3D7_0508900	conserved Plasmodium protein		370.2

IMC, inner membrane complex; PVM, parasitophorous vacuole membrane; SP, signal peptide; TM, transmembrane; Y2H, yeast-two-hybrid.

Roughly 60% (76/126) of the SSH genes have been detected in the currently available *P. falciparum* gametocyte proteomes, while for 40% there is as yet no protein evidence (Table 2) [9,12,13,22]. Almost identical numbers apply to the 113 orthologs of the rodent malaria parasite *P. berghei*; the 13 *P. falciparum* genes without *P. berghei* orthologs include amongst others the three *etramp* genes, *pfs16* and *resa*.

**Table 2:** **Comparison of genes identified by SSH with *P. falciparum *and *P. berghei *gametocyte proteome data**

			**Pf**	***P. falciparum *GC**				***P. berghei *male/female GC**							**Total**	
**PF_ID**	**Gene Ontology**	**MW**	**Troph**	**I & II**	**IV & V**	**V**	**I.II.IV.V**	**FG1**	**FG 3**	**FG (1+3)**	**MG1**	**MG3**	**MG (1+3)**	**FG +MG**	**FG.MG.PF**	**PB_ID**
PF3D7_0210600	signaling	53.7	0	0	0	0	0	0	0	0	0	0	0	0	0	PBANKA_030750
PF3D7_0214600	signaling	204.3	0	0	0	0	0	0	0	0	0	0	0	0	0	PBANKA_031140
PF3D7_0302100	signaling	157.4	2	3	1	7	11	0	0	0	0	0	0	0	11	PBANKA_040110
PF3D7_0303900	signaling	23.2	0	4	4	11	19	0	0	0	0	0	0	0	19	PBANKA_040250
PF3D7_0515300	signaling	255.9	0	0	0	0	0	0	0	0	0	0	0	0	0	PBANKA_111490
PF3D7_0530200	signaling	59.8	0	0	0	3	3	0	0	0	0	0	0	0	3	PBANKA_124460
PF3D7_0624000	signaling	55.3	17	20	2	24	46	6	13	19	11	10	21	40	86	PBANKA_112290
PF3D7_0818200	signaling	30.2	18	21	14	25	60	11	20	31	9	11	20	51	111	PBANKA_071260
PF3D7_0927200	signaling	189.7	0	13	2	5	20	0	0	0	0	0	0	0	20	PBANKA_082800
PF3D7_1004300	signaling	131.7	3	5	1	0	6	0	0	0	0	0	0	0	6	PBANKA_120260
PF3D7_1113100	signaling	25.1	1	2	1	3	6	0	0	0	0	0	0	0	6	PBANKA_093450
PF3D7_1230900	signaling	111.5	0	0	0	0	0	0	0	0	0	0	0	0	0	PBANKA_144560
PF3D7_1233600	signaling	146.3	0	4	0	8	12	0	0	0	0	0	0	0	12	PBANKA_144820
PF3D7_1238100	signaling	266.4	7	9	3	9	21	2	6	8	8	7	15	23	44	PBANKA_145260
PF3D7_1238900	signaling	58.9	0	0	0	0	0	0	0	0	0	0	0	0	0	PBANKA_145340
PF3D7_1356800	signaling	475.7	0	0	0	0	0	0	0	0	0	0	0	0	0	PBANKA_113310
PF3D7_1358600	signaling	118.3	0	0	0	0	0	0	0	0	0	0	0	0	0	PBANKA_113490
PF3D7_1403900	signaling	35.2	6	9	5	12	26	×	×	×	×	×	×	×	26	×
PF3D7_1433400	signaling	682.5	0	0	2	0	2	0	0	0	0	0	0	0	2	PBANKA_101150
PF3D7_1442200	signaling	118.7	0	0	1	0	1	0	0	0	0	0	0	0	1	PBANKA_130610
PF3D7_1463900	signaling	127.1	0	0	0	0	0	0	0	0	0	0	0	0	0	PBANKA_132710
PF3D7_1464600	signaling	170.5	23	31	0	3	34	0	0	0	0	0	0	0	34	PBANKA_132800
PF3D7_0420300	cell cycle & gene expr.	400.2	0	0	0	0	0	0	0	0	0	0	0	0	0	PBANKA_052170
PF3D7_0513600	cell cycle & gene expr.	129.2	10	29	0	10	39	0	0	0	0	0	0	0	39	PBANKA_111330
PF3D7_0615400	cell cycle & gene expr.	323.2	0	0	0	0	0	0	0	0	0	0	0	0	0	PBANKA_123010
PF3D7_0622900	cell cycle & gene expr.	237.4	0	0	0	0	0	0	0	0	0	0	0	0	0	PBANKA_112180
PF3D7_0811300	cell cycle & gene expr.	200.2	0	2	1	2	5	0	0	0	0	0	0	0	5	PBANKA_142620
PF3D7_0827800	cell cycle & gene expr.	283.6	0	0	1	0	1	0	0	0	0	0	0	0	1	PBANKA_070290
PF3D7_1011800	cell cycle & gene expr.	131.6	41	22	6	17	45	4	11	15	14	0	14	29	74	PBANKA_121020
PF3D7_1018500	cell cycle & gene expr.	12.5	0	0	0	0	0	0	0	0	0	0	0	0	0	PBANKA_050270
PF3D7_1019000	cell cycle & gene expr.	280.8	7	0	0	0	0	0	0	0	0	0	0	0	0	PBANKA_050320
PF3D7_1034900	cell cycle & gene expr.	101.2	21	31	0	49	80	6	0	6	3	0	3	9	89	PBANKA_051870
PF3D7_1103800	cell cycle & gene expr.	392.0	7	12	0	23	35	0	0	0	0	0	0	0	35	PBANKA_094310
PF3D7_1105500	cell cycle & gene expr.	20.1	1	0	0	0	0	0	0	0	0	0	0	0	0	PBANKA_094140
PF3D7_1115200	cell cycle & gene expr.	94.3	0	0	0	0	0	0	0	0	0	0	0	0	0	PBANKA_093250
PF3D7_1347500	cell cycle & gene expr.	42.2	18	15	30	22	67	11	0	11	7	10	17	28	95	PBANKA_136030
PF3D7_1360900	cell cycle & gene expr.	44.8	8	11	11	12	34	×	×	×	×	×	×	×	34	×
PF3D7_1417200	cell cycle & gene expr.	519.8	5	6	0	6	12	0	0	0	0	0	0	0	12	PBANKA_102550
PF3D7_1445900	cell cycle & gene expr.	60.0	16	25	3	21	49	0	0	0	1	0	1	1	50	PBANKA_130970
PF3D7_1469700	cell cycle & gene expr.	24.6	0	2	0	3	5	0	0	0	0	0	0	0	5	PBANKA_133290
PF3D7_0422300	IMC, cytoskeleton	49.7	11	25	13	30	68	0	0	0	0	0	0	0	68	PBANKA_052270
PF3D7_0503400	IMC, cytoskeleton	13.7	6	5	1	8	14	0	0	0	0	1	1	1	15	PBANKA_110310
PF3D7_0515700	IMC, cytoskeleton	51.8	3	0	0	7	7	1	0	1	0	0	0	1	8	PBANKA_111530
PF3D7_0708000	IMC, cytoskeleton	103.6	0	0	2	12	14	0	0	0	1	0	1	1	15	PBANKA_080520
PF3D7_0724900	IMC, cytoskeleton	222.6	0	0	0	0	0	0	0	0	0	0	0	0	0	PBANKA_062240
PF3D7_0918000	IMC, cytoskeleton	44.6	14	12	1	23	36	0	0	0	0	0	0	0	36	PBANKA_081900
PF3D7_1020200	IMC, cytoskeleton	99.1	0	0	0	0	0	0	0	0	0	0	0	0	0	PBANKA_050430
PF3D7_1246200	IMC, cytoskeleton	41.2	23	28	13	29	70	12	19	31	6	6	12	43	113	PBANKA_145930
PF3D7_1246400	IMC, cytoskeleton	23.5	1	0	0	3	3	0	0	0	0	0	0	0	3	PBANKA_145950
PF3D7_1251200	IMC, cytoskeleton	69.0	1	0	0	0	0	0	0	0	0	0	0	0	0	PBANKA_146410
PF3D7_1351700	IMC, cytoskeleton	151.2	0	0	1	0	1	0	0	0	0	0	0	0	1	PBANKA_136440
PF3D7_0102200	proteostasis	126.5	33	3	1	0	4	×	×	×	×	×	×	×	4	×
PF3D7_0215400	proteostasis	110.8	0	0	1	0	1	0	0	0	0	0	0	0	1	PBANKA_031210
PF3D7_0306200	proteostasis	41.6	4	7	0	8	15	0	0	0	0	0	0	0	15	PBANKA_040460
PF3D7_0708400	proteostasis	86.2	54	60	17	82	159	7	0	7	8	10	18	25	184	PBANKA_080570
PF3D7_0807500	proteostasis	29.5	11	11	2	20	33	3	8	11	4	2	6	17	50	PBANKA_122310
PF3D7_0815800	proteostasis	214.7	0	3	0	0	3	0	0	0	0	0	0	0	3	PBANKA_071500
PF3D7_0818900	proteostasis	73.9	45	46	33	57	136	34	40	74	31	28	59	133	269	PBANKA_071190
PF3D7_0917900	proteostasis	72.4	45	40	42	51	133	24	46	70	16	16	32	102	235	PBANKA_081890
PF3D7_1136400	proteostasis	107.1	0	3	0	11	14	1	0	1	0	0	0	1	15	PBANKA_091220
PF3D7_1466300	proteostasis	132.8	23	26	3	34	63	4	9	13	4	0	4	17	80	PBANKA_132970
mal_mito_2	metabolism general	53.0	0	0	0	0	0	0	0	0	0	0	0	0	0	PBANKA_MIT0002
PF3D7_0104400	metabolism general	62.5	0	1	1	3	5	0	0	0	0	0	0	0	5	PBANKA_020870
PF3D7_0403200	metabolism general	69.4	0	0	0	0	0	0	0	0	0	0	0	0	0	PBANKA_100080
PF3D7_0511800	metabolism general	69.1	36	29	2	40	71	2	0	2	0	0	0	2	73	PBANKA_111140
PF3D7_0801900	metabolism general	552.0	0	0	1	0	1	0	0	0	0	0	0	0	1	PBANKA_122830
PF3D7_0806800	metabolism general	123.0	11	9	4	29	42	0	0	0	0	3	3	3	45	PBANKA_122380
PF3D7_0922200	metabolism general	44.8	25	16	0	4	20	1	2	3	0	0	0	3	23	PBANKA_082310
PF3D7_1444800	metabolism general	40.1	21	23	11	35	69	17	23	40	22	22	44	84	153	PBANKA_130860
PF3D7_0102500	cell surface & PVM	181.2	0	0	0	0	0	0	0	0	0	0	0	0	0	PBANKA_133270
PF3D7_0202500	cell surface & PVM	11.5	1	0	0	0	0	×	×	×	×	×	×	×	0	×
PF3D7_0406200	cell surface & PVM	16.7	3	9	34	17	60	×	×	×	×	×	×	×	60	×
PF3D7_0423700	cell surface & PVM	10.2	0	5	0	2	7	×	×	×	×	×	×	×	7	×
PF3D7_0707300	cell surface & PVM	103.6	3	0	0	0	0	0	0	0	0	0	0	0	0	PBANKA_080450
PF3D7_0731500	cell surface & PVM	174.6	0	0	0	0	0	0	0	0	0	0	0	0	0	PBANKA_133270
PF3D7_0930300	cell surface & PVM	195.7	77	0	3	0	3	0	0	0	0	0	0	0	3	PBANKA_083100
PF3D7_1021900	cell surface & PVM	267.4	11	26	4	31	61	0	0	0	0	0	0	0	61	PBANKA_112590/ PBANKA_141230
PF3D7_1028700	cell surface & PVM	58.1	0	0	1	11	12	0	0	0	0	0	0	0	12	PBANKA_051280
PF3D7_1033200	cell surface & PVM	38.9	11	16	0	4	20	×	×	×	×	×	×	×	20	×
PF3D7_1035300	cell surface & PVM	141.1	0	0	1	0	1	×	×	×	×	×	×	×	1	×
PF3D7_1035700	cell surface & PVM	80.3	10	0	0	0	0	×	×	×	×	×	×	×	0	×
PF3D7_1103500	cell surface & PVM	67.3	0	0	0	0	0	0	0	0	0	0	0	0	0	PBANKA_094340
PF3D7_1218800	cell surface & PVM	39.6	0	0	2	5	7	0	0	0	0	0	0	0	7	PBANKA_143440
PF3D7_1228600	cell surface & PVM	86.6	23	0	0	0	0	0	0	0	0	0	0	0	0	PBANKA_144330
PF3D7_1335300	cell surface & PVM	382.9	0	0	0	0	0	0	0	0	0	0	0	0	0	PBANKA_000220 + 10 more
PF3D7_0206200	others	62.7	0	0	0	2	2	0	0	0	0	0	0	0	2	PBANKA_030390
PF3D7_0207700	others	108.7	11	0	0	0	0	0	0	0	0	0	0	0	0	PBANKA_030480
PF3D7_0311600	others	84.0	0	0	0	5	5	0	0	0	0	0	0	0	5	PBANKA_040960
PF3D7_0320700	others	21.0	0	0	0	8	8	0	0	0	0	0	0	0	8	PBANKA_121780
PF3D7_0403800	others	83.4	0	0	0	0	0	0	0	0	0	0	0	0	0	PBANKA_100150
PF3D7_0709900	others	274.0	0	0	0	0	0	0	0	0	0	0	0	0	0	PBANKA_122050
PF3D7_0817600	others	112.2	0	0	0	0	0	0	0	0	0	0	0	0	0	PBANKA_071320
PF3D7_0930000	others	66.8	0	0	0	0	0	0	0	0	0	0	0	0	0	PBANKA_083070
PF3D7_1027800	others	44.2	17	9	14	18	41	0	0	0	0	0	0	0	41	PBANKA_051190
PF3D7_1033800	others	52.3	0	0	0	0	0	0	0	0	0	0	0	0	0	PBANKA_051760
PF3D7_1103100	others	13.0	2	2	5	4	11	1	0	1	0	0	0	1	12	PBANKA_094360
PF3D7_1207700	others	43.4	0	0	0	0	0	0	0	0	0	0	0	0	0	PBANKA_060620
PF3D7_1358800	others	17.3	8	6	4	9	19	8	5	13	1	0	1	14	33	PBANKA_113510
PF3D7_1408600	others	25.1	8	8	7	12	27	2	5	7	2	0	2	9	36	PBANKA_103390
PF3D7_1457000	others	47.6	0	0	0	0	0	2	0	2	0	1	1	3	3	PBANKA_132070
PF3D7_0202400	unknown	141.9	27	0	0	0	0	×	×	×	×	×	×	×	0	×
PF3D7_0411000	unknown	179.7	0	0	0	0	0	0	0	0	0	0	0	0	0	PBANKA_061240
PF3D7_0417400	unknown	822.4	0	0	1	0	1	0	0	0	0	0	0	0	1	PBANKA_071950
PF3D7_0508900	unknown	370.2	0	2	0	0	2	0	0	0	0	0	0	0	2	PBANKA_110850
PF3D7_0601900	unknown	14.7	0	0	0	1	1	×	×	×	×	×	×	×	1	×
PF3D7_0704100	unknown	425.3	0	0	0	0	0	0	0	0	0	0	0	0	0	PBANKA_080180
PF3D7_0730800.1	unknown	32.1	5	0	0	0	0	×	×	×	×	×	×	×	0	×
PF3D7_0904200	unknown	34.0	0	1	0	0	1	0	0	0	0	0	0	0	1	PBANKA_041720
PF3D7_1021700	unknown	830.2	0	0	0	1	1	1	0	1	0	0	0	1	2	PBANKA_050590
PF3D7_1023000	unknown	48.9	0	0	0	0	0	0	0	0	0	0	0	0	0	PBANKA_050720
PF3D7_1024800	unknown	171.1	4	33	15	33	81	0	0	0	0	0	0	0	81	PBANKA_050900
PF3D7_1026100	unknown	23.9	0	0	0	0	0	0	0	0	0	0	0	0	0	PBANKA_051030
PF3D7_1026600	unknown	196.5	0	0	0	0	0	×	×	×	×	×	×	×	0	×
PF3D7_1114600	unknown	39.6	0	0	0	0	0	0	0	0	0	0	0	0	0	PBANKA_093310
PF3D7_1124200	unknown	49.3	0	0	0	7	7	0	0	0	1	0	1	1	8	PBANKA_092410
PF3D7_1126700	unknown	110.1	0	1	0	0	1	0	0	0	0	0	0	0	1	PBANKA_092170
PF3D7_1225600	unknown	93.1	0	1	0	0	1	0	0	0	0	0	0	0	1	PBANKA_144050
PF3D7_1230100	unknown	61.5	0	0	0	0	0	0	0	0	0	0	0	0	0	PBANKA_144480
PF3D7_1239400	unknown	22.0	0	0	0	0	0	0	0	0	0	0	0	0	0	PBANKA_145390
PF3D7_1316700	unknown	73.4	0	0	0	0	0	0	0	0	0	0	0	0	0	PBANKA_141520
PF3D7_1321000	unknown	80.6	0	0	0	0	0	0	0	0	0	0	0	0	0	PBANKA_141930
PF3D7_1362600	unknown	32.1	0	0	0	0	0	0	0	0	0	0	0	0	0	PBANKA_113860
PF3D7_1438800	unknown	84.0	0	0	0	2	2	0	0	0	0	0	0	0	2	PBANKA_130270
PF3D7_1439600	unknown	22.6	0	6	0	10	16	1	0	1	0	0	0	1	17	PBANKA_130350
PF3D7_1451200	unknown	176.2	0	0	0	0	0	0	0	0	0	0	0	0	0	PBANKA_131490
PF3D7_1452400	unknown	107.5	0	0	0	0	0	0	0	0	0	0	0	0	0	PBANKA_131610

PF troph, peptide hits for gametocyte-less *P. falciparum* trophozoites [12,13]; I & II, stage I & II *P. berghei* gametocyte [12,13]; V, stage V *P. falciparum* gametocytes [9]; I.II.IV.V, sum of all *P. falciparum* gametocyte data; FG1, peptide hits from highly purified female *P. berghei* gametocyte sample 1 [22]; FG3, peptide hits from highly purified female *P. berghei* gametocytes sample 3 [22]; FG(1+3), sum of both; MG1, peptide hits from highly purified male *P. berghei* gametocyte sample 1 [22]; MG3, peptide hits from highly purified male *P. berghei* gametocyte sample 3 [22]; MG(1+3), sum of both; FG+MG, total number peptide hits from all four *P. berghei* gametocyte samples; total FG.MG.PF, total number of *P. falciparum* &*P. berghei* gametocyte peptide hits.

The *P. berghei* orthologs of the identified *P. falciparum* genes were compared with a global gametocyte transcriptome analysis of *P. berghei* DOZI and CITH gene deletion mutants; destabilized mRNAs of these mutants are candidates for translational repression in non-activated gametocytes [19,20]. A total of 11 genes (8.7%) were identified as candidates regulated by DOZI and CITH (Table 3); eight of those lack any gametocyte protein evidence. One of the destabilized genes was identified as a single peptide hit while the remaining two destabilized genes had 19 and 74 peptide hits in total. A number of 16 additional genes were destabilized in the combined DOZI and CITH datasets indicating they could also be under translational control (data not shown). The data comparison indicates that the majority of genes identified via SSH, which change in transcript expression levels during gametocyte activation, are not translationally controlled in non-activated gametocytes, while many such genes have been identified to function in zygote to ookinete transformation [23]. In conclusion, gene regulation appears to be important for the early events of gametocyte activation, i.e. for gametogenesis and fertilization, while translational repression particularly plays a role for the expression of proteins important for ookinete development.

**Table 3:** **Genes identified by SSH with *P. berghei *orthologs showing destabilization in the absence of DOZI and CITH**

***P. falciparum *gene ID**	**P**	**Protein**	**Predicted feature**	**MW**	***P. berghei *ortholog gene ID**	**DOZI**	**CITH**
PF3D7_1011800	74	QF122 antigen	KH domain type 1, RNA binding	131.6	PBANKA_121020	1.4	1.1
PF3D7_0724900	0	kinesin-like protein		222.6	PBANKA_062240	2.4	1.2
PF3D7_1033800	0	plasmepsin VII	SP, 1 TM, aspartyl protease	52.3	PBANKA_051760	2.1	1.3
PF3D7_1316700	0	conserved Plasmodium protein	SP	73.4	PBANKA_141520	2.1	1.4
PF3D7_1321000	0	conserved Plasmodium protein		80.6	PBANKA_141930	1.8	1.6
PF3D7_0303900	19	conserved Plasmodium protein	SP, phosphatidyl-ethanolamine-binding protein PEBP domain	23.2	PBANKA_040250	2.2	1.8
PF3D7_1103500	0	CPW-WPC family protein	SP, CPW-WPC domain	67.3	PBANKA_094340	1.3	2.3
PF3D7_1115200	0	SET domain protein, SET7	Chromatin regulator	94.3	PBANKA_093250	2.5	2.3
PF3D7_1207700	0	blood stage antigen 41–3 precursor	SP	43.4	PBANKA_060620	2.5	2.8
PF3D7_0904200	1	conserved Plasmodium protein	SP, 1 TM	34	PBANKA_041720	1.9	3.2
PF3D7_1362600	0	conserved Plasmodium protein	Interacts with splicing factor 3B subunit 2	32.1	PBANKA_113860	3.4	3.5

P, total number of peptides identified in *P. falciparum* and *P. berghei* gametocytes [9,12,13,22]; DOZI and CITH, log_2_ values of transcript abundance WT compared to DOZI-KO, or WT compared to CITH KO [20].

**Figure 2:**
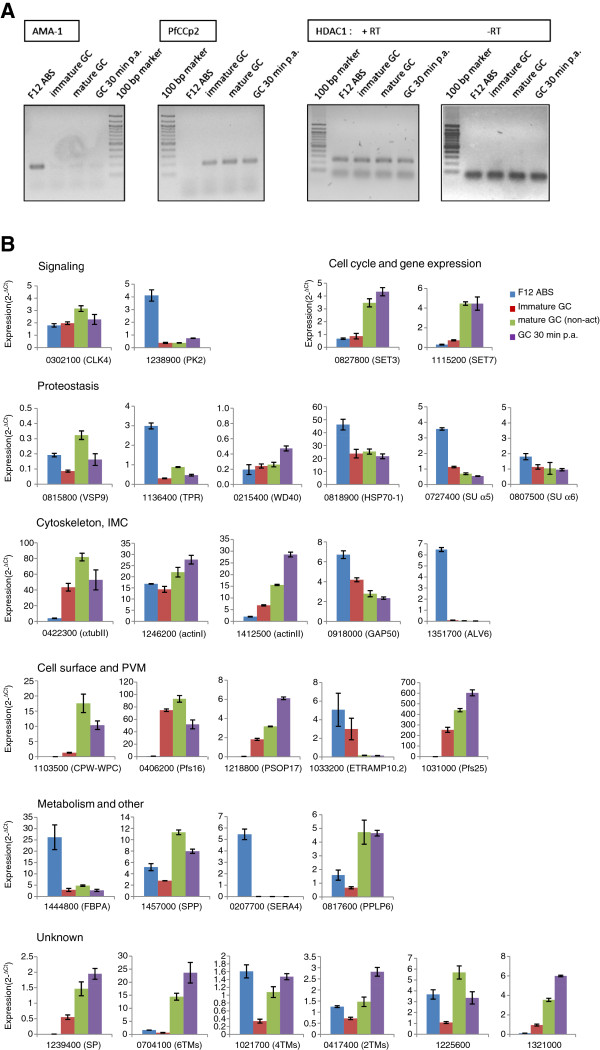
**Changes in transcript expression levels during the formation and activation of *P. falciparum *gametocytes.** (**A**) Diagnostic RT-PCR demonstrated the presence of stage-specific transcript in the asexual blood stages of the gametocyte-less strain F12 (F12 ABS) as well as in immature (stage III/IV) and mature (stage V) non-activated gametocytes (GC) and in gametocytes at 30 min p.a. in vitro. *ama-1*, marker for ABS; *pfccp2*, marker for GC. Transcript of gene *hdac1* was detected in all stages investigated, while no *hdac1*-specific PCR product was detected in samples lacking reverse transcriptase (RT). (**B**) Real-time RT-PCR analysis showed changes of transcript expression of 27 of the SSH-identified genes between ABS, immature and mature non-activated GCs and in GCs at 30 min p.a. in vitro. Transcript expression levels were calculated by the 2^-ΔCt^ method; the threshold cycle number (Ct) was normalized with the Ct of the gene encoding for seryl tRNA synthetase as reference.

We used real-time RT-PCR analyses to confirm the transcript changes in gametocytes during activation for a subset of the identified genes and to determine, if these genes become up- or down-regulated in their expression during gametocyte activation. Total RNA was isolated from immature (stages III and IV) and mature (stage V) gametocytes of *P. falciparum* strain NF54 and from activated gametocytes at 30 min post-activation (p.a.). Furthermore, we isolated RNA from mixed asexual blood stages of the gametocyte-less *P. falciparum* strain F12. Initially, the synthesized cDNA of each sample was tested for its stage-specificity by diagnostic RT-PCR, using primers for the asexual blood stage gene *ama-1*[24,25] and for the gametocyte-specific gene *pfccp2*[26]. The tests confirmed that no *pfccp2* transcript was present in the F12 sample, while no *ama-1* expression was detected in the purified gametocyte samples of strain NF54 (Figure 2A, left). The absence of *ama-1* signals in the gametocyte samples and the absence of *pfccp2* in the F12 asexual blood stage sample further showed that these were devoid of any contamination by genomic DNA (gDNA). An additional test for gDNA contamination was performed by using primers specific for the gene *hdac1* (histone deacetylase 1; PF3D7_0925700). In all parasite samples, i.e. in samples of F12 asexual blood stages as well as in NF54 immature, mature and activated gametocytes, *hdac1* transcript was present (Figure 2A, right), as shown by diagnostic RT-PCR. In sample preparations lacking reverse transcriptase, on the other hand, no *hdac1*-specific PCR bands were detected.

We chose 27 genes from the SSH analysis for comparison of transcript abundance, with representatives from all ontology groups. The gene *pfs16* was selected as an internal control, because it is known to be highly expressed in gametocytes throughout development, while it is absent in the gametes [7,27,28]. Furthermore two sexual stage-specific genes, *actin II* and *pfs25*, served as external controls. Pfs25 is expressed in vesicular structures during gametocyte maturation and relocates to the surface of macrogametes following activation. Pfs25 is subsequently present on the parasite surface until the ookinete stage [16,29-32]. Actin II is a sexual stage-specific actin isomer, and in *P. berghei* actin II was reported to play a role during microgametogenesis [33,34]. In addition, the gene *su α5*, encoding for subunit (SU) *α5* of the α-ring of the proteasome core particle [35], was included in the investigations.

Transcript expression levels were measured via real-time RT-PCR and calculated by the 2^-ΔCt^ method [36,37] in which the threshold cycle number (Ct), was normalized to the Ct of the endogenous control gene encoding for *P. falciparum* seryl tRNA synthetase (PF3D7_0717700) as reference gene [38,39]. Transcript levels with 2^-ΔCt^ values below 0.5 were considered negligible. Real-time RT-PCR revealed transcription in mature gametocytes for 22 out of the 27 genes. Out of these, 15 genes showed increased transcript expression in gametocytes compared to asexual blood stage parasites (Figure 2B).

Seven genes were identified, i.e. PF3D7_0827800 (*set3*), Pf3D7_1246200 (*actin I*), PF3D7_1218800 (*psop17*), PF3D7_1239400, PF3D7_0704100, PF3D7_0417400, and PF3D7_1321000, for which transcript levels were increased in activated gametocytes as compared to asexual blood stages, immature gametocytes and non-activated gametocytes (Figure 2B), indicating that these genes may play an important role downstream of gametocyte activation in the mosquito midgut. SET3 was previously described to accumulate in male gametocytes, where it contributes to a prompt entry and execution of S/M phases during microgametogenesis [40]. SET domains are assigned to chromatin dynamics and are often found in histone methyltransferases, thus they play a role in the epigenetic control of gene regulation. The *P. falciparum* genome encodes for at least nine SET-domain-containing proteins which exhibit five different types of substrate specificities [41].

Actin I was described as part of the plasmodial motor complex (reviewed in [42]) and was recently also reported to be present in gametocytes [33,43]. Furthermore, PSOP17 (putative secreted ookinete protein 17) was previously reported to be expressed in mature gametocytes of *P. falciparum*[9,12] and in ookinetes of *P. berghei*[44]. In addition, transcript expression of the two control genes, PF3D7_1031000 (*pfs25*) and PF3D7_1412500 (*actin II*) was strong in gametocytes compared to asexual blood stage parasites and increased during gametocyte activation (Figure 2B).

Eight genes had an increased transcript expression in gametocytes compared to asexual blood stage parasites, and transcript levels either remained constant or decreased following activation. These include PF3D7_0302100 (*clk4*), PF3D7_1115200 (*set7*), PF3D7_0422300 (*α-tubulin II*), PF3D7_1103500 (encoding for a CPW-WPC protein), PF3D7_406200 (*pfs16*), PF3D7_1457000 (*spp*), PF3D7_0817600 (encoding for a MAC/PF domain, here termed PPLP6) and PF3D7_1225600 (Figure 2B). The mRNA splicing kinase CLK4 (also termed SRPK1) is expressed in the asexual blood stages and gametocytes of *P. falciparum*[45,46] and a gene knock-out of the orthologous protein in *P. berghei* failed to exflagellate upon gametocyte activation [47]. The gene *set7* encodes for one of the nine SET-domain-containing proteins (see above). Transcript of *set7* is destabilized in the absence of DOZI and CITH (compare with Table 1), pointing at a translational repression of *set7* in gametocytes and a role in epigenetic control mechanisms of the ookinete.

Pfs16 is a transmembrane protein of the gametocyte PVM [12,27,48,49]. After the PVM has ruptured during the egress of the activated gametocyte from the host cell, Pfs16 is not detectable any longer in the sexual stage parasites [7] (see below), explaining the decrease of transcript following activation. Furthermore, α-tubulin II represents a tubulin isoform that is expressed in asexual blood stage parasites, in gametocytes and in microgametes [50-52]. Targeted gene modification studies in *P. berghei* indicated that α-tubulin II plays an important role for microgametogenesis [53]. The fact that *α-tubulin II* transcript levels decrease during gametogenesis lets us conclude that the α-tubulin II filaments needed for the formation of the microgametes are already present in the mature gametocytes prior to activation.

The presenilin-like signal peptide peptidase (SPP) was previously reported to be involved in merozoite invasion and cleavage of the erythrocyte cytoskeletal protein band 3 [54]. A recent study, however, disagreed with these findings, and reported that SPP is an ER-resident protease required for growth of the erythrocytic stages [55]. SPP is considered a potential drug target of liver and blood stage parasites [55-57]. The combined data indicate that SPP is present in the liver, blood and gametocyte stages, pointing to a role of SPP in multiple life-cycle stages of *Plasmodium*.

The gene product of PF3D7_0817600 comprises a MAC/PF domain, similar to the MAC/PF domains of the previously described plasmodial perforin-like proteins PPLP1-5 [58] (reviewed in [8]). We therefore termed the protein PPLP6 in this study. Furthermore, the gene product of PF3D7_1103500 is a member of a family of nine secreted proteins with cysteine-rich CPW-WPC domains [59] (reviewed in [60]). The CPW-WPC domain is a conserved domain of about 61 residues in length that exhibits six well-conserved cysteine residues and six well-conserved aromatic sites. The functions and life cycle expression patterns of the plasmodial CPW-WPC proteins are hitherto not known.

Three genes were predominantly expressed in the asexual blood stages and show a resurgence in transcript expression in gametocytes during maturation, i.e. PF3D7_1238900 (*pk2*), PF3D7_1136400 (encoding for a tetratrico peptide repeat region; TPR), and PF3D7_1021700. The calcium/calmodulin-dependent protein kinase PK2 was hitherto only investigated in the asexual blood stages of *P. falciparum*[61,62] and protein expression in gametocytes has not yet been investigated. The gene product of PF3D7_1136400 comprises a TPR domain, which is known to mediate protein-protein interactions and the assembly of multiprotein complexes (reviewed in [63]), but the function of the plasmodial TPR domain protein is not yet known. While transcript levels of PF3D7_1136400 increase during gametocyte development, a drop following activation was observed.

For four genes, PF3D7_0818900 (*hsp70-1*), PF3D7_080700 (*su α6*)*,* PF3D7_0807500 (*gap50*), and PF3D7_1444800 (encoding for fructose 1,6 bisphosphate aldolase, FBPA) expression was high in asexual blood stages and decreased during gametocyte development and following activation (Figure 2B). SU α6 is a component of the plasmodial proteasome, a proteolytic complex composed of more than 33 SUs that is responsible for the degradation and recycling of ubiquitinated proteins. As an external control we thus investigated the transcript levels of another α-ring component, *su α5*, and revealed similar changes in the transcript levels of *su α5* in asexual blood stage parasites and gametocytes before and after activation (Figure 2B).

The genome of *P. falciparum* encodes for a variety of chaperones, including heat shock proteins (HSPs) of the HSP70, HSP90 and DnaJ/HSP40 families (reviewed in [64]). HSP70-1 was previously described to be located in the cytoplasm and nucleus of the parasite blood stages [65] as well as in the PV, pointing to a role in protein transport to the erythrocyte [66]. Another proposed function of HSP70-1 includes the protein trafficking to the apicoplast [67,68], indicating that the chaperone has several essential functions and is important for multiple life-cycle stages. It remains to be elucidated, if other of the identified HSPs have more specific functions during parasite transmission from human to mosquito. Interestingly in this context, HSP90 belongs to a family of evolutionarily conserved chaperones which has been suggested as a capacitor for morphological evolution because reduction of its function results in phenotypic variation in *Drosophila*[69]. In addition, HSP90 displays multiple roles in stress adaptation and development, including spermatogenesis, oogenesis and embryogenesis in insects [70].

The transmembrane protein GAP50 is part of the IMC of the parasite invasive stages, including the ookinete [42,71]. Here, it links myosin with the outer membrane of the IMC and thus contributes to gliding motility of the parasite. Recently, GAP50 was also described as a component of the gametocyte IMC [43,72,73], which appears to be important for the stability of the crescent-shaped cell. Noteworthy, transcript of the *P. berghei* orthologous protein was shown to co-precipitate with DOZI and CITH (G.R. Mair, unpublished observations), indicating that *gap50* is translationally repressed in gametocytes. Thus, the release of the translational repression and onset of protein synthesis during gametocyte activation might cause the detected decrease in the *gap50* transcript level.

FBPA is an enzyme of glycolysis, and in *Plasmodium* is also reported to be part of the motor complex, here linking TRAP to actin I [71,74]. Noteworthy in this context, during gametocytogenesis, malaria parasites shift from glycolysis towards mitochondrial respiration [75], which might explain the decrease in FBPA expression in gametocytes compared to asexual blood stage parasites.

Transcript expression for five genes, PF3D7_0825800 (*vsp9*), PF3D7_0215400 (encoding for a WD40 motif), PF3D7_1351700 (*alv6*), PF3D7_1033200 (*etramp10.2*), and PF3D7_0207700 (*sera4*), is negligible in gametocytes. The gene PF3D7_0815800 encodes for a homolog to the yeast vacuolar sorting protein VSP9 with a predicted function in protein-protein interactions, while PF3D7_0215400 encodes for a protein with a WD40 motif. WD-repeat proteins are a large family found in all eukaryotes, implicated in a variety of functions ranging from signal transduction and transcription regulation to cell cycle control and apoptosis. The WD40 motifs act as a site for protein-protein interaction, and proteins containing WD40 repeats are known to serve as platforms for the assembly of protein complexes or mediators of transient interplay among other proteins (reviewed in [76]). WD-repeat proteins of *P. falciparum* have hitherto been described as receptors for protein kinase C, and as components of the myosin-driven motor complex [77,78]. Furthermore, ALV6 is a member of the alveolin family, which comprises seven proteins in *P. falciparum*, associated with the membranous sacs of the IMC [79]. The fact that ALV6 expression decreases during gametocytogenesis is fairly surprising, considering that gametocytes possess an IMC (see above). It has to be elucidated, if the expression of any other alveolin is up-regulated in gametocytes.

The early transcribed membrane proteins (ETRAMPs) are proteins of the plasmodial PVM [80,81]. In *P. falciparum* the proteins were shown to form complexes with the PVM protein exported protein 1 (EXP-1) [82]. To date, ETRAMP10.2 transcripts were found in *P. falciparum* trophozoites and the mixed asexual stages and the liver stages of *P. yoelii*[80,81]. Because EXP-1 is also present in the gametocyte PVM [7], the expression of some of the ETRAMPs in these stages can be expected. It thus remains to be elucidated, if the other two SSH-identified ETRAMPs, ETRAMP2 and ETRAMP4, might play a specific role for gametocytes. Expression of serine repeat antigen SERA4 in the asexual blood stages has previously been described [83]. The SERA family comprises nine proteins with functions in asexual blood stage growth and host cell egress (reviewed in [8,84]).

**Figure 3:**
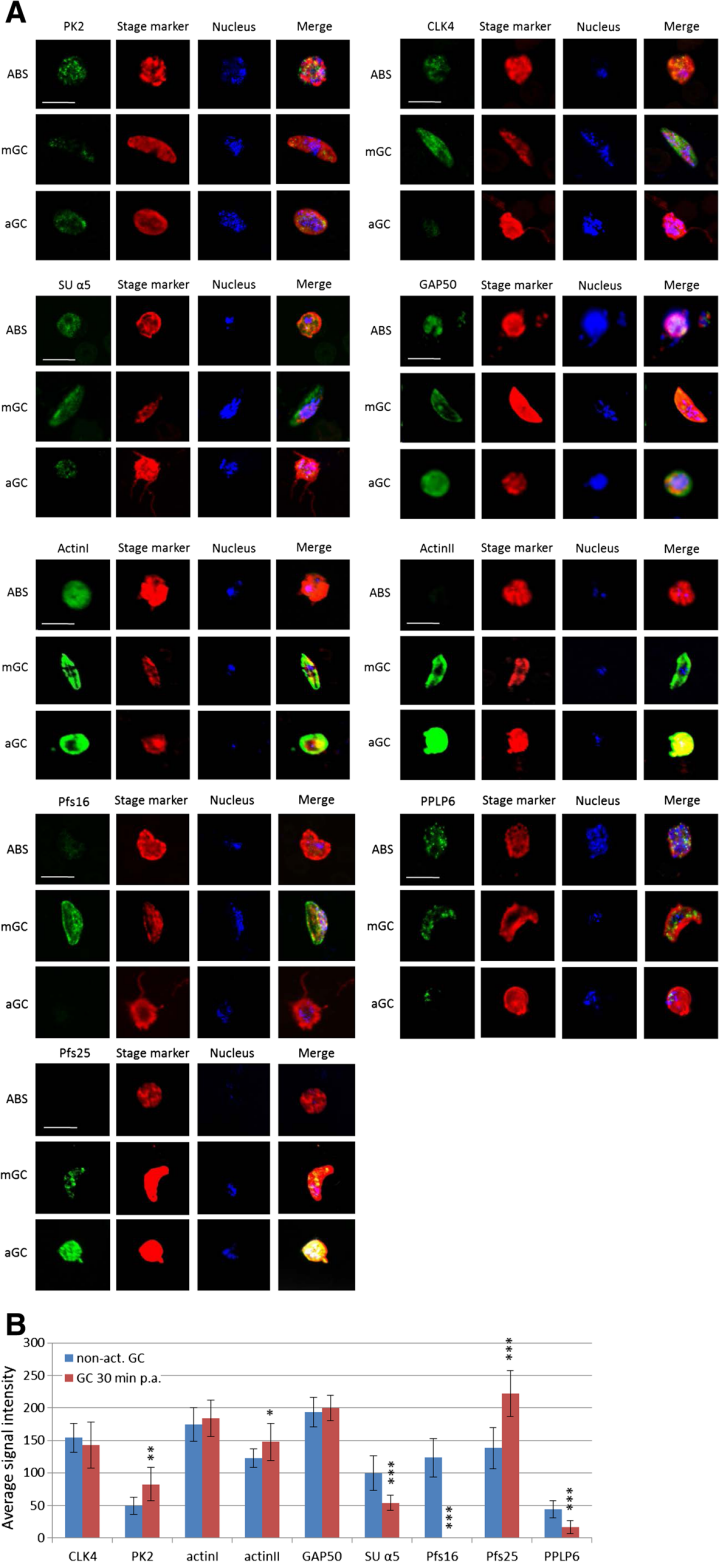
**Changes in protein expression during formation and activation of *P. falciparum *gametocytes.** (**A**) Immunofluorescence assays, using specific antibodies, detected the proteins of interest (in green) in asexual blood stage (ABS) parasites, in gametocytes (GC) and in gametocytes at 30 min p.a. (aGC). The parasite stages were highlighted with antibodies against stage-specific markers (in red; MSP1 for ABS; Pfs230 or Pfs25 for GCs and aGCs). Nuclei were highlighted by Hoechst nuclear stain (in blue). Bar, 5 μm. (**B**) Diagram showing the average signal intensity of the immunolabeled proteins in gametocytes before and at 30 min p.a. in vitro. Measurements were performed on 20 plotted cells per setting via quantitative confocal microscopy. Mean ± SD. *p < 0.05;**p < 0.01; ***p < 0.001 (student’s t-test).

For selected genes we subsequently investigated the expression changes on the protein level. Antibodies against the proteins PK2, CLK-4, actin I and actin II, GAP50, proteasome SU α5, Pfs16, Pfs25 and PPLP6 were used to immunolabel the respective proteins in samples of *P. falciparum* F12 asexual blood stages, non-activated NF54 gametocytes and gametocytes at 30 min p.a. via indirect immunofluorescence assay (Figure 3A). Asexual blood stage parasites were highlighted by MSP-1 labeling; gametocytes were highlighted by labeling of Pfs25 or Pfs230, respectively. The abundance of the respective proteins in gametocytes before and after activation was quantified by measuring the average fluorescence signal intensity of a total number of 20 plotted cells per setting (Figure 3B). A significant up-regulation in the expression of PK2, actin II and Pfs25 following gametocyte activation was confirmed (Figure 3A, B). While PK2 is also expressed in asexual blood stage parasites, actin II and Pfs25 cannot be detected in these stages. On the other hand, CLK4, proteasome SU α5, and PPLP6 were detected in asexual blood stage parasites and gametocytes during maturation, but the proteins were down-regulated in gametocytes at 30 min p.a. (Figure 3B). While labeling of CLK4 and SU α5 revealed a homogenous expression of these proteins in the parasite cytoplasm and nucleus, PPLP6-labeling exhibited a punctuated expression (Figure 3A). Noteworthy, the PPLP6 labeling disappeared after the gametes have fully egressed from the enveloping erythrocyte. A role of the perforin PPLP1 was assigned to rupturing the PVM during host cell egress by the Apicomplexan parasite *Toxoplasma gondii*[85], and a recent study reported the involvement of PPLP2 in the lysis of the erythrocyte membrane during the exflagellation of male *P. berghei* gametocytes [86]. We thus hypothesize that the plasmodial PPLP6 might also play a role in the rupture of the enveloping membranes of the activated gametocytes, explaining its disappearance during gametogenesis. Similarly, the PVM-based protein Pfs16 was not present in gametocytes at 30 min p.a., since at this time point the parasites have egressed from the enveloping erythrocyte and the PVM was destroyed [7]. Furthermore, in agreement with the real-time RT-PCR results, actin I is present in both, asexual blood stages and gametocytes, and a minor up-regulation in the activated gametocytes was detected (Figure 3A, B).

Interestingly, we demonstrated a slight up-regulation in the protein expression of GAP50 at 30 min p.a., although a decrease in transcript levels was described during this process. This phenomenon can be explained by the fact that translationally repressed *gap50* transcript is present in the non-activated gametocytes (see above), and that the release of repression during activation leads to rapid translation and in consequence to a loss in transcript abundance and an increase in protein abundance. It is worth mentioning in this context, that we recently showed a relocation of GAP50 from the gametocyte IMC to the plasma membrane during gametogenesis. Once on the gamete surface, GAP50 binds the human complement regulator protein factor H from the blood meal, which enables the extracellular parasite to prevent lysis by the human complement [43].

In summary we demonstrate that the majority of genes, which are regulated in their expression levels during gametocyte activation, have functions assigned to signaling, cell cycle and gene regulation, and proteostasis, or that they are cytoskeletal or cell surface proteins. The regulated expression of proteins involved in signaling and cell cycle control is on the one hand important for the signaling pathways that result in the induction of gametogenesis, once temperature drop and XA are perceived by the parasite [47] (reviewed in [6,87]; on the other hand gametocyte activation releases the parasites from cell cycle arrest, subsequently leading to three rounds of rapid DNA replication during microgametogenesis [88].

Furthermore, induction of gametogenesis following gametocyte activation results on the one hand in the destruction of the gametocyte IMC and relocation of cytoskeletal proteins [7,33,34,72,73], on the other hand in a drastic turn-over of surface proteins (reviewed in [6]). The molecular changes in the composition of cytoskeletal and surface proteins are important for stage conversion during gametogenesis and for adaptation of the parasite to the insect host and also promote the development of the parasite midgut stages, like the ookinete. The changes in protein expression during parasite transmission from human to insect further explain the importance of chaperones and assembly proteins. The proteasome on the other hand appears to play only a minor role during gametogenesis. We hypothesize that proteins which lost their function, once the parasite has entered the mosquito vector, are only degraded after the gametes have developed and after fertilization has occurred, thus after the phase of rapid stage conversions has finished.

Noteworthy, the SSH analysis identified a number of unknown proteins with transmembrane domains and/or signal peptides, and for six of them, high transcript levels were shown in mature and activated gametocytes. For three of the genes, expression in the gametocytes was higher as compared to asexual blood stage parasites, and these genes might encode for proteins important for gametocyte development or gametogenesis.

## Conclusions

The here presented data on the changes in the *P. falciparum* transcriptome during gametogenesis provide first insights into the regulated gene expression occurring while the malaria parasite passes the initial phase of transmission to the mosquito. Our findings form the basis for further studies on genes important for malaria transmission and support the identification of proteins involved in the development of the mosquito midgut stages, which might lead to the discovery of new transmission blocking targets.

## Methods

### Antibodies

The following antibodies were used in this study: mouse antisera against Pfs16 and Pfs230 (kindly provided by Kim Williamson, Loyola University Chicago), proteasome SU α5 [35] and actin II [34], as well as rabbit antisera against GAP50 (kindly provided by Julian Rayner, Sanger Institute England; [89]), Pfs230 (against the immunogenic region C) [90], MSP-1 and Pfs25 (BEI Resources, Manassas). Mouse antisera against actin I, PPLP6, PK2, and CLK4 were generated for this study (see below).

### Parasite culture

Gametocyte-less F12 strain and gametocyte-forming NF54 isolate of *P. falciparum* were cultivated in vitro in RPMI 1640 medium complemented with 10% heat-inactivated human serum as described [91]. As soon as stage II gametocytes started to emerge in the NF54 culture, the culture medium was supplemented with 50 mM N-acetyl glucosamine (GlcNac) for approximately 5 days to kill the asexual blood stages [92]. The gametocyte culture was then maintained in normal culture medium without GlcNac until immature (stage III and IV) or mature stage V gametocytes were harvested and enriched by Percoll gradient purification [93]. The mature stage V gametocytes were divided into two aliquots, and one aliquot was subsequently incubated in gametogenesis activating solution (1.67 mg/ml glucose; 8 mg/ml NaCl; 1 mg/ml Tris–HCl (pH 8.2) [94], containing 100 μM XA for 30 min at RT.

### Construction of a subtracted cDNA library using the SSH method

Total RNA was isolated from Percoll-enriched stage V gametocytes before and at 30 min p.a., using Trizol reagent (Invitrogen, Karlsruhe, Germany). Subsequently, mRNA was isolated from total RNA using the Oligotex mRNA Mini Kit (Qiagen, Hilden, Germany) according to manufacturer’s instructions, and the SSH analysis was conducted as previously described [21,95] using the PCR-Select cDNA Subtraction Kit from Clontech (Mountain View, CA, USA), according to the manufacturer’s protocol. Briefly, 220 ng of purified mRNA were reverse-transcribed into cDNA using a cDNA synthesis primer; subsequently double-stranded cDNA was generated and digested with RsaI. The double-stranded cDNA from activated gametocytes was ligated in separate aliquots to adaptor 1 or adaptor 2R and were denaturated at 98°C for 90 s and then hybridized at 68°C for 8 h with a 30-fold excess of double-stranded cDNA of non-activated gametocytes. Subsequently, both samples were mixed together with a 10-fold excess of freshly denatured double-stranded cDNA from non-activated gametocytes and hybridized at 68°C for 16 h. The sample was then subjected to two rounds of suppression PCR with PCR-primer 1 and nested primers supplied with the kit. PCR amplifications were performed in a total volume of 25 μl using a PCR cycler (Biometra, Göttingen, Germany) with a heated lid and the 5^′^Prime PCR Extender System (5^′^Prime, Hamburg, Germany). An initial adapter extension at 75°C for 5 min was followed by a denaturation step at 93°C for 30 s and by 27 cycles of denaturation at 93°C for 15 s, annealing at 66°C for 30 s, and extension at 72°C for 90 s. A final 7-min 72°C step was added to allow complete extension of the products. The secondary PCR was performed with nested primer 1 and 2R on 1 μl of the primary PCR products for 15 cycles with an initial denaturation step at 93°C for 1 min, followed by denaturation at 93°C for 15 s, annealing at 68°C for 30 s and extension at 72°C for 90 s, plus a final extension step at 72°C for 7 min. Resulting PCR products of the secondary subtractive PCR were purified using the NucleoSpin Extract II kit (Macherey Nagel, Düren, Germany), ligated into the pGEM-T easy vector (Promega, Mannheim, Germany) and transformed into NEB 5-alpha competent *E. coli* (New England BioLabs, Frankfurt, Germany). The library was plated on 2×YT agar plates containing 100 μg/ml ampicillin and incubated at 37°C for 16 h.

### Colony PCR, sequencing and computer analysis of cDNA sequence data

A preliminary colony PCR screen on 20 colonies was performed with vector-specific primers, i.e. T7-promotor: 5^′^- TAATACGACTCACTATAGGG-3^′^ and SP6: 5^′^-ATTTAGGTGACACTATAG- 3^′^ (purchased from Thermo electron, Waltham, MA, USA), using the following conditions: denaturation at 95°C for 5 min followed by 30 cycles of denaturation at 95°C for 15 s, annealing at 43°C for 20 s, and extension at 72°C for 60 s. A final 7-min 72°C step was added to allow complete extension of the products. The colony PCR showed that 75% of clones contained an insert in the vector. Subsequently, 288 randomly picked clones were screened for the presence of the vector. Plasmid isolation of 141 positively screened colonies was performed with the Fast Plasmid Mini kit (Eppendorf, Hamburg, Germany) and the inserts were sequenced (GATC Biotech, Konstanz, Germany; sequences listed in the Additional file 1: Table S1). Sequences were used to identify similar sequences of the National Center for Biotechnology Information databases using BLASTX program (BLASTX 2.2.13; http://www.ncbi.nlm.nih.gov/BLAST/) and to predict signal sequences, transmembrane regions and features, using the PlasmoDB program (http://www.plasmodb.org) [96].

### RNA isolation and real-time RT-PCR

Total RNA was isolated from the mixed asexual F12 cultures, enriched immature NF54 gametocytes (stage III and IV), non- activated mature gametocytes and gametocytes at 30 min p.a. using the Trizol reagent (Invitrogen) according to the manufacturer’s protocol. RNA preparations were treated with RNAse-free DNAse I (Qiagen) to remove gDNA contamination, followed by phenol/chloroform extraction and ethanol precipitation. All RNA samples had A260/A280 ratios higher than 2.1. Two μg of each total RNA sample were used for cDNA synthesis using the SuperScript III First-Strand Synthesis System (Invitrogen), following the manufacturer’s instructions. Controls without reverse transcriptase were used to investigate potential gDNA contamination by diagnostic PCR using *hdac1*-specific primers. RNA quality was further verified for contamination by monitoring transcripts of stage-specific genes, i.e. *ama1* and *pfccp2*, by diagnostic RT-PCR (for primer sequences, see Additional file 2: Table S2).

Primers for real-time RT-PCR were designed using the Primer 3 software (http://frodo.wi.mit.edu/primer3/) and were initially tested on gDNA in conventional PCR with the same conditions subsequently used for real-time RT-PCR (for primer sequences, see Additional file 2: Table S2). Primers for the reference gene encoding for seryl tRNA synthetase were obtained from [38]. The PCR products were subsequently separated by agarose gel electrophoresis. Primers with high specificity were further validated by testing amplification efficiency on 10-fold dilutions of gDNA using real-time RT-PCR followed by melt curve analysis. Primers with low specificity, efficiency and poor melt curves were redesigned.

Real-time RT-PCR measurements were performed using the Bio-Rad CFX96 Real-Time Detection System. Reactions were prepared in triplicate in a total volume of 20 μl using the maxima SyBR green qPCR master mix (Thermo Scientific, Bonn, Germany) with 20 ng of cDNA and primer concentrations of 0.3 μM. The following PCR cycling conditions were used: an initial denaturation step at 95°C for 10 min, followed by 40 cycles of denaturation at 95°C for 15 s and annealing/extension at 60°C for 60 s, followed by a final extension at 95°C for 10 s and a final melt-curve analysis of 55-95°C. Controls without template and without reverse transcriptase were included in all real-time RT-PCR experiments. Transcript expression levels were calculated by the 2^-ΔCt^ method [36,37], using the endogenous control gene encoding for *P. falciparum* seryl tRNA synthetase (PF3D7_0717700) as reference [38,39]. Control samples lacking reverse transcriptase during cDNA synthesis were routinely included during each run and exhibited an average threshold cycle number of 39.

### Recombinant protein expression and production of mouse antisera

Recombinant proteins for CLK4, PK2, and actin I were expressed as fusion proteins with a GST-tag using the pGEX 4 T-1 vector (Amersham Biosciences, Freiburg, Germany), PPLP6 recombinant proteins were expressed as maltose binding protein-tagged fusion proteins using vector pIH902 (kindly provided by K. Williamson, Chicago; [90]). DNA was amplified by PCR using gene-specific primers (for primer sequences, see Additional file 2: Table S2). Recombinant proteins were expressed in BL21 (DE3) RIL cells according to the manufacturer’s protocol (Invitrogen). Recombinant proteins were isolated as either inclusion bodies (PK2, actin I) as described [16] or affinity-purified (CLK4 and PPLP6) using glutathione sepharose (GE Healthcare, Munich, Germany) and amylose resin (New England Biolabs), respectively, according to the manusfacturers´protocols. Specific immune sera were generated by immunizing 6-week-old female NMRI mice (Charles River Laboratories, Sulzfeld) with 100 μg recombinant protein emulsified in Freund’s incomplete adjuvant (Sigma-Aldrich), followed by a boost after 4 weeks. Sera were collected 10 days after the boost. The housing and handling of the animals followed the guidelines of the animal welfare committee of Lower Franconia.

### Indirect immunofluorescence assay and signal strength quantification

Asexual F12 blood stage parasites, non-activated NF54 gametocytes and gametocytes at 30 min p.a. were air dried on glass slides and fixed for 10 min in −80°C methanol. For membrane permeabilization and blocking of non-specific binding, fixed cells were incubated for 30 min in 0.01% saponin/0.5% BSA/PBS and 1% neutral goat serum (Sigma-Aldrich) in PBS. Preparations were then successively incubated for 1.5 h each at 37°C with the primary antibody diluted in 0.01% saponin/0.5% BSA/PBS. Binding of primary antibody was visualized using fluorescence-conjugated goat anti-mouse or anti-rabbit secondary antibodies (Alexa Fluor 488 or Fluor 596; Molecular Probes, Karlsruhe, Germany) diluted in 0.01% saponin/0.5% BSA/PBS. Nuclei were highlighted by incubating the specimens with Hoechst nuclear stain 33342 (Molecular Probes) for 1 min. Cells were mounted with anti-fading solution AF2 (Citifluor Ltd., Leicester, UK) and sealed with nail varnish. Digital images were taken using a Leica TCS SP5 confocal laser scanning microscope and processed using Adobe Photoshop CS software. For quantitative evaluation of the average signal intensity, digital images of 20 randomly selected mature and activated gametocytes were taken using a Zeiss LSM 510 confocal laser scanning microscope under the same optimal laser scanning microscopy settings for each antibody. The average fluorescence signal intensity of each cell, as indicated by a line edging the respective cell, was measured by LSM 510 image software and recorded.

## Competing interests

The authors of this manuscript declare no competing interests.

## Authors’ contribution

CJN and MS conducted the experiments. SK, TB, MNA and CW generated antibodies. RF, AV and GP designed the experiments. CJN, MS, GRM, JW, and GP contributed with data analyses. CJN and GP contributed with writing the manuscript. All authors read and approved the manuscript.

## Supplementary Material

Additional file 1 Colony sequences of SSH analysis. Click here for file

Additional file 2 Sequences of primers used in this study. Click here for file
