# Supplementary material for: Changes in the transcriptome of the malaria parasite Plasmodium falciparum during the initial phase of transmission from the human to the mosquito
Source: BMC Genomics. 2013 Apr 15;14:256. doi: 10.1186/1471-2164-14-256 (PMC3640944; doi:10.1186/1471-2164-14-256)
Supplement: Additional file 2 — Sequences of primers used in this study. [file 1471-2164-14-256-S2.pdf]

Additional table 2 Sequences of primers used in this study.

PlasmoDB gene ID	Name/feature	Sense primer (5'---3')	Anti-sense primer (5'---3')
**RT-PCR primers**			
**PF3D7_0302100**	**CLK-4**	GCCATACGATCCGTCTGTTT	CATGCATGCAAAGGACCATA
**PF3D7_1238900**	**PK2**	ATCCGAATGATCCAAGCTCA	TTACCTCGGAAAGGTGGGTA
**PF3D7_0827800**	**SET3**	TGGTGAGAAGTAGCGACGAG	TTACGACACCTCTCCCTTCG
**PF3D7_1115200**	**SET7**	TGGGTTCCAGAACCTGATAAA	ATGATGGCCGAAAGCATTAT
**PF3D7_0815800**	**VPS9**	TGTTACCTTGTGCTGATGAA	CAGCATTCGCTACTTTTTCT
**PF3D7_1136400**	**TPR**	TGTTGATCAAAATATCGTTCATGT	AAAAAGTTCTTGAACTAGTTGCTCTTT
**PF3D7_0215400**	**WD40**	GCCAAATCACCAGCAAAAAT	GCTGATGGGGACATATGGTT
**PF3D7_0818900**	**HSP70-1**	GAATCGGTTTGTGCTCCAAT	CAACTGTTGGTCCACTTCCA
**PF3D7_0727400**	**Proteasome SU α5**	GTGCGATGAGTGGTTTGATG	AAGCAACTCCGAATGGTCTG
**PF3D7_0807500**	**Proteasome SU α6**	GTTTTTGTGCGGGATATCGT	GCTTCAATTGCCAAAATGGT
**PF3D7_0422300**	**Alpha -tubulinII**	GGCCATCTCCTCAAGTATCG	TCAACATTCAAAGCACCATCA
**PF3D7_1246200**	**actinI**	AAAGAAGCAGCAGGAATCCA	TTGATGGTGCAAGGGTTGTA
**PF3D7_1412500**	**actinII**	GGAATGTCCGGGTTTACACA	TCTTTCAGGTGGAGCAATGA
**PF3D7_0918000**	**GAP50**	TCTTTGGGTGATTGGGGTAA	TCCATGCTGGATCATTTAAGC
**PF3D7_1351700**	**ALV6**	TCAAAACATCTACTCGCACCA	CCCGAATGAAAATTCGTACC
**PF3D7_1103500**	**CPW-WPC**	ATTCAGTGAAGGCACAAATC	CGTCTGGAATATCATCTGCT

**PF3D7_0406200**	**Pfs16**	CAAGGTGGACTATCTCAAGG	TTTATCATCATCTGCGTTCTT
**PF3D7_1218800**	**PSOP17**	ATTACCATCGGCAAAACCAT	CCAATTCCTCCAAATCCAGA
**PF3D7_1033200**	**ETRAMP10.2**	CTGCCCTTGTCGTTACAGGT	CCGAATTTACGGTACGTGCT
**PF3D7_1031000**	**Pfs25**	AATGCGAAAGTTACCGTGGA	CAAGCGTATGAAACGGGATT
**PF3D7_1444800**	**FBPA**	TGTACCACCAGCCTTACCAG	TTCCTTGCCATGTGTTCAAT
**PF3D7_1457000**	**SPP**	CAGTATGCTTGGTTTAGGAGA	TAAAGAAGAGCTGGTTGAGG
**PF3D7_0207700**	**SERA4**	ATACTGGTTCCCAAGGAGAT	TTTCTGGTGGTGCTAATTCT
**PF3D7_0817600**	**PPLP6**	GGGAGTACCACCACCCTTTT	GTCGTTTTATGGGCAGCACT
**PF3D7_1239400**	**SP**	TGTTCAAAAACAACAGTTCCAA	TCCATTGGTTTGCTAGGTGA
**PF3D7_0704100**	**6 TMs**	TCAGACGATCACGCTATTGG	TCCCGTCTGACTATTTCCTTG
**PF3D7_1021700**	**4 TMs**	AAACGAAAGGATGCCACAAG	CGCTTGGGCTGCTACTACAT
**PF3D7_0417400**	**2 TMs**	TGCAAAGACGAAACATTCCA	TGTCCGTATGCTTCAAGGAA
**PF3D7_1225600**		TGATGAAAAGTCCAAAGGGAAT	TTTTCCGGTCCTCATCACTT
**PF3D7_1321000**		AGAAGATCATGTACATCTTCACAAA	TCATCATTTGATGCAAGTACGAG
**PF3D7_0925700**	**HDAC1**	TGGTCTTTTTCAATTTCAGCAA	GCTTCCTCAACACCATCTCC
**PF3D7_1133400**	**AMA1**	GGATTATGGGTCGATGGA	GATCATACTAGCGTTCTT
**PF3D7_1455800**	**PfCCp2**	TCGGATGGAGAATCCGTT	GTATCCCATGTCTTGTGA
**PF3D7_0717700**	**Seryl tRNA synthetase**	AAGTAGCAGGTCATCGTGGTT	TTCGGCACATTCTTCCATAA
**Primers used for recombinant protein expression**			
**PF3D7_1238900**	**PK2**	<u>GAATTC</u>ATGGAGAAAAGATATCAGCAA	<u>GAATTC</u>TTAGGGAGATCTTCTAGAACACTTAT

**Pf3D7_0302100**	**CLK-4**	TAGGATCCTCCAATAACAGCAACAGT	TACCCGGGTTATTTGGTAATCCCTTCCGCTTT
**PF3D7_1246200**	**Actin I**	ATGGATCCGCAGGAGTTTCAGGAGATGAT	TAGCGGCCGCTTAGTGGATTCCTGCTGCTTC
**PF3D7_0817600**	**PPLP6**	ATGAATTCGCAATAGAAATGAAAAGTGATGAAAA	TAGAATTCTTACCCACTAAATCGAGAAAAAGC

Restriction sites are underlined.
